# Desorption Electrospray Ionization Mass Spectrometry Imaging Illustrates the Quality Characters of Isatidis Radix

**DOI:** 10.3389/fpls.2022.897528

**Published:** 2022-06-15

**Authors:** Li-Xing Nie, Lie-Yan Huang, Xin-Ping Wang, Lin-Feng Lv, Xue-Xin Yang, Xiao-Fei Jia, Shuai Kang, Ling-Wen Yao, Zhong Dai, Shuang-Cheng Ma

**Affiliations:** ^1^National Institutes for Food and Drug Control, National Medical Products Administration, Beijing, China; ^2^WHO Collaborating Center for Herbal Medicine (CHN-139), Beijing, China; ^3^Hebei University of Chinese Medicine, Shijiazhuang, China; ^4^Shaoxing Institutes for Food and Drug Control, Shaoxing, China; ^5^Waters Corporation, Beijing, China

**Keywords:** mass spectrometry imaging (MSI), Isatidis Radix, herbal medicine, quality, desorption electrospray ionization (DESI), quadrupole-time-of-flight mass spectrometry (Q-TOF/MS), orthogonal partial least squares discrimination analysis (OPLS-DA)

## Abstract

For a long history, herbal medicines have made significant contributions to human health all around the world. However, the exploration of an effective approach to illustrate their inner quality remains a challenge. So, it is imperative to develop new methods and technologies to characterize and identify quality markers of herbal medicines. Taking Isatidis Radix, the dried root of *Isatis indigotica* as an example, desorption electrospray ionization (DESI), in combination with quadrupole-time-of-flight mass spectrometry (Q-TOF/MS), was applied in this work for the first time to reveal the comprehensive spatial distribution of metabolites and, further, to illustrate quality characters of this herbal medicine. After simple pretreatment, 102 metabolites including alkaloids, sulfur-containing compounds, phenylpropanoids, nucleosides, amino acids, organic acids, flavonoids, phenols, terpenes, saccharides, peptides, and sphingolipids were characterized, some of which were successfully localized and visualized in the transverse section of the root. Based on the ion images, samples with different quality characters were distinguished unambiguously by the pattern recognition method of orthogonal partial least squares discrimination analysis (OPLS-DA). Simultaneously, 11 major influencing components exerting higher ion intensities in superior samples were identified as the potential quality markers of Isatidis Radix. Desorption electrospray ionization (DESI) mass spectrometry imaging (MSI), together with chemometric analysis could not only improve the understanding of the plant biology of herbal medicines but also be beneficial in the identification of quality markers, so as to carry out better quality control of herbal medicines.

## Introduction

Herbal medicine plays an active role in disease prevention and treatment from ancient times till now. As a typical case, Isatidis Radix, the dried root of *Isatis indigotica*, is well acknowledged for its effects on treating fever, pestilence, sore throat, seasonal toxin, papule caused by warm toxin, macula, scarlatina, mumps, swelling abscess, erysipelas, and erysipelas facialis (Chinese Pharmacopoeia Commission, 2020). To guarantee the efficacy of Isatidis Radix, it is of paramount importance to conduct quality control on it. The Chinese Pharmacopeia (Chinese Pharmacopoeia Commission, 2020) determines the content of (*R, S*)-goitrin to evaluate the quality of Isatidis Radix. But the single-marker approach is not comprehensive for the herb with a multicomponent system. Traditional experiences for quality evaluation of Isatidis Radix were generally established according to its morphological features, but modern studies mostly focused on the contents of chemical components, making chemical analysis come apart with empirical identification. Therefore, it is necessary to explore a new strategy to build a direct link between the quality characters and chemical profiles of this herbal medicine.

In recent years, the new technique of mass spectrometry imaging (MSI) has been proven as a powerful tool to explore the spatial distribution of molecules in biomaterials including cell (Mcmahon and Lechene, 2021; Meng et al., 2021), plant (Hu et al., 2021; Tong et al., 2021), animal (Yang et al., 2020; Sisley Emma et al., 2022), and human being (Kip et al., 2021). Thanks to its *in situ*, label-free, and untargeted capabilities, MSI offers the unprecedented spatio-chemical information, which is lost in routine homogenization-based approaches. Compared with the most sophisticated MSI techniques such as secondary ion mass spectrometry (SIMS) (Karas et al., 1985), desorption electrospray ionization (DESI) holds its distinct features in simple procedure, relatively low cost, and flexibility to be combined with various mass analyzers. The unique operating principle (Takáts et al., 2004) gives DESI imaging a tempting character that it has a low requirement for sample preparation. There is no need for the application of matrix. What's more, the section doesn't have to be very thin. The above advantages may explain why DESI MSI is popular in the investigation on herbal medicines lately, which is difficult to slice and section. With no or less pretreatment, various kinds of chemicals in herbal medicines could be visualized in their native tissues by DESI MSI (Srimany et al., 2016; Mohana Kumara et al., 2019). In addition, pharmacological and toxicological mechanisms of components isolated from herbal medicines could also be illustrated by this technique (He et al., 2015; Wang et al., 2020). Apart from herbal materials, DESI-MSI was also adopted in the rapid semi-quantification of herbal products (Qu et al., 2020; Sun et al., 2020). Although playing an active role in the above arenas, DESI MSI has scarcely been applied to link the chemical profiling and the quality characters of herbal medicines.

In our previous work, a MALDI MSI-based approach was developed to reveal the distributions of phytochemicals in Isatidis Radix. In total, 118 compounds were identified, and samples from different habitats were differentiated successfully (Nie et al., 2021). In this study, desorption electrospray ionization combined with quadrupole-time-of-flight mass spectrometry (DESI-Q-TOF/MS) was applied for the first time to illustrate the quality-related morphological characters of Isatidis Radix, providing a deeper insight into the understanding of the quality of the herbal medicine.

## Materials and Methods

### Chemicals and Materials

The LC-MS grade methanol, ethanol, acetonitrile, formic acid, 0.1% (w/v) poly(*L*-lysine) hydrobromide solution, and gelatin were obtained from Sigma-Aldrich (Shanghai, China). Analytical grade ammonium acetate, chloral hydrate, phloroglucinol, iodine, sulfuric acid, and ethanol were obtained from Sinopharm Chemical Reagent Co., Ltd (Beijing, China). The optimum cutting temperature (OCT) compound was obtained from Leica (Nussloch, Germany). Water was purified by employing a Milli-Q filtration system (Millipore, Bedford, USA). Information for the reference standards could be found in Table 1. Sixty-four batches of Isatidis Radix were purchased from herb markets in the provinces of Hebei, Gansu, Sichuan, and Heilongjiang, China. The origin of each sample was authenticated as the dried root of *Isatis tinctoria* L. (Brassicaceae) by Associate Professor Shuai Kang in accordance with the Chinese Pharmacopeia, edition 2020. For future reference, the voucher specimens (S1–S64) were deposited in NIFDC, Beijing, China.

**Table 1:** Compound name, chemical class, and supplier of the reference standards.

**Compound name**	**Chemical class**	**Supplier**
*L*-tyrosine	Amino acids	National Institutes for Food and Drug Control (Beijing, China), NIFDC for short
*L*-arginine	Amino acids	NIFDC
*L*-proline	Amino acids	NIFDC
Indirubin	Alkaloids	NIFDC
Indigo	Alkaloids	NIFDC
Oleamide	Alkaloids	NIFDC
3-Indoleacetic acid	Alkaloids	Shanghai Yuanye Bio-Technology Co., Ltd. (Shanghai, China), Yuanye for short
4-Hydroxyindole-3-carboxaldehyde	Alkaloids	Yuanye
3-Indoleformic acid	Alkaloids	Yuanye
Isatin	Alkaloids	Yuanye
Indole-3-acetamide	Alkaloids	Yuanye
Deoxyvasicinone	Alkaloids	Yuanye
Isoindigo	Alkaloids	Yuanye
3-Indoxyl-β-D-glucoside	Alkaloids	Yuanye
4-Hydroxyquinazoline	Alkaloids	Yuanye
3-Indoleacetonitrile	Alkaloids	Yuanye
3-Formylindole	Alkaloids	Yuanye
Methyl-indole-3-carboxylate	Alkaloids	Yuanye
Adenine	Nucleosides	NIFDC
Adenosine	Nucleosides	NIFDC
Guanosine	Nucleosides	NIFDC
Uridine	Nucleosides	NIFDC
Guanine	Nucleosides	NIFDC
Inosine	Nucleosides	NIFDC
Uracil	Nucleosides	NIFDC
2′-*O*-methyladenosine	Nucleosides	Yuanye
Cytidine	Nucleosides	NIFDC
2′-Deoxyinosine	Nucleosides	Yuanye
(*R, S*)-goitrin	Sulfur-containing compounds	NIFDC
Epiprogoitrin	Sulfur-containing compounds	Yuanye
Progoitrin	Sulfur-containing compounds	Yuanye
Sinigrin	Sulfur-containing compounds	Shanghai Standard Technology Co., Ltd (Shanghai, China)
Maleic acid	Organic acids	Shanghai Standard Technology Co., Ltd (Shanghai, China)
Citric acid	Organic acid	Shanghai Standard Technology Co., Ltd (Shanghai, China)
Guaiacol	Organic acids	NIFDC
α-Linolenic acid	Organic acids	NIFDC
Salicylic acid	Organic acids	NIFDC
Benzoic acid	Organic acids	NIFDC
Palmitic acid	Organic acids	NIFDC
Stearic acid	Organic acids	NIFDC
Linoleic acid	Organic acids	NIFDC
Palmitoleic acid	Organic acids	NIFDC
Oleic acid	Organic acids	NIFDC
Malic acid	Organic acids	NIFDC
*n*-Octanoic acid	Organic acids	NIFDC
Syringic acid	Organic acids	Yuanye
Coniferin	Phenylpropanoids	Yuanye
Syringin	Phenylpropanoids	Yuanye
Lariciresinol	Phenylpropanoids	Yuanye
Isolariciresinol	Phenylpropanoids	Yuanye
Dihydroconiferyl alcohol	Phenylpropanoids	Yuanye
Eucalyptol	Terpenes	NIFDC
Limonene	Terpenes	NIFDC
Vomifoliol	Terpenes	Yuanye
Sucrose	Saccharides	NIFDC
D-fructose	Saccharides	NIFDC
D-glucose	Saccharides	NIFDC
Luteolin-6-*C*-glucoside	Flavonoids	NIFDC
Isovitexin	Flavonoids	Yuanye
Isoscoparin	Flavonoids	Yuanye

### Quality Characterization Based on Macroscopy and Microscopy

To investigate the quality characters of Isatidis Radix, macroscopic and microscopic features of the herb were observed. For macroscopic characterization, features including the texture, the color of surface, the depth of longitudinal wrinkle, and the existence of roothead were described and compared. For each batch of sample, the lengths of the longest, medium, and shortest individuals, and the diameters at the middle of the thickest, medium, and thinnest individuals were measured and recorded. Then, the root with the medium size of each batch was cut from the middle, and the character of the transverse section was observed and photographed using a digital single-lens reflex (DSLR) camera (Canon 5D4, Tokyo, Japan). For microscopic characterization, a 1-cm section was cut axially from the middle of the root with the medium size of each batch, which was embedded in the OCT compound before freezing at −13°C. Next, the frozen root was sectioned into a 30-μm slice with the tape at −13°C by employing a cryomicrotome (Leica CM 1950, Nussloch, Germany). The microscopic features of the cross-section of Isatidis Radix were observed *via* a microscope (Olympus BX51, Tokyo, Japan) after permeabilizing with chloral hydrate solution. To examine the lignified cells and the starches, the section was stained by phloroglucinol solution and sulfuric acid, and iodine test solution, respectively. An automatic digital slide scanning imaging system (Zeizz Axiocan.z1, Jena, Germany) was used to collect the images. Finally, the quality characters for Isatidis Radix were proposed based on morphological investigation combined with literature research.

### Sample Preparation for DESI MSI

The dried root was cut from the middle to obtain a 0.5-cm-long piece, which was embedded with 0.1 g/ml gelatin solution and frozen at −20°C. For cryosectioning, the frozen sample was axially fixed on the sample holder of a cryomicrotome (Leica CM1950, Nussloch, Germany), using OCT compound as the adhesive. Cross-sections of 100 μm thickness were obtained at−18°C and adhered on a glass slide with the double-sided conductive tape (3M, St. Paul, MN, USA).

### UHPLC-Q-Orbitrap MS Analysis

An Ultimate 3000 ultra-high performance liquid chromatography system coupled with Quadruple-Enactive Orbitrap (UHPLC-Q-Orbitrap) high-definition tandem mass spectrometer (Thermo Fisher Scientific, Waltham, USA) was adopted to identify the chemical constituents in extracts of Isatidis Radix. Xcalibur software (Thermo Fisher Scientific, Waltham, USA) was used for instrument control and parameter setting. The sample was pulverized and shifted through a 60-mesh sieve, and the powder was extracted with mixtures of methanol and water at different ratios. Chromatographic separation was performed on a Waters CORTECS UPLC T_3_ column (2.1 mm × 100 mm, 1.6 μm) of 35°C. The following binary gradient consisting of methanol (A) and water (B), both with 10 mM ammonium acetate at a flow rate of 0.2 ml/min was applied as follows: 0 min 2% A, 40 min 40% A, 45 min 68% A, 55 min 95% A, 65 min 95% A, 70 min 2% A, and 80 min 2% A. The sample inject volume was 1 μl. The full-MS and data-dependent scan (dd-MS^2^) under both ESI^+^ and ESI^−^ modes was used for Q-Orbitrap MS/MS data acquisition with the mass spectrometer parameters as follows: The data acquisition range was *m*/*z* 50–1,500 Da. The spray voltage was 3.5 kV for ESI^+^ mode and 3.0 kV for ESI^−^ mode. The sheath gas and auxiliary gas were operated at flow rates of 35 arb and 10 arb, respectively. The temperature of the capillary was 350°C and that of the auxiliary gas heater was 350°C, and the S-lens RF level was 50.

### DESI Mass Spectrometry Imaging

Mass imaging of the tissue was carried out on a Q-TOF mass spectrometer equipped with the DESI source (Waters Xevo G2-XS, Milford, USA). The parameters were set as follows: mass range, *m/z* 50–1,200; ion source temperature, 150°C; capillary voltage, 4.0 kV; spray solvent, MeOH-H_2_O (95:5, containing 0.1% formic acid); spray solvent speed, 2 μl/min; spray gas (N_2_) pressure, 0.45 MPa; incident spray angle, 75°; collection angle, 10°; X and Y pixel sizes, 80 μm; and raster speed, 200 μm/s. A calibration solution of 0.2 mg/L leucine encephalin in methanol (Waters, Milford, MA, USA) was used as the lockmass for high-resolution mass spectra. To measure the positive and negative ions separately, two slices were prepared for each sample. To validate the identification results of metabolites in Isatidis Radix, all the reference standards were prepared at a concentration of 10 μg/ml with 70% (v/v) methanol. Then, 0.5 μl of each of the reference solutions was applied on a glass slide and dried in the air. Ultimately, the spots of the reference standards were scanned by the same parameters of the sample.

### Data Analysis

All images were reconstructed by linear smoothing and displayed in absolute intensity after total ion current (TIC) normalization. Screening of the ions was carried out by the aid of the database including more than 400 components in Isatidis Radix, which was constructed by the authors using UNIFI software (Waters, Milford, MA, USA) and other commercial databases. Further identification was based on the accurate mass to charge ratio with reference to the reference standards and/or the literature and databases and MS/MS information obtained by UHPLC-Q-Orbitrap MS analysis. The region of interest (ROI) was selected from the whole scanned sector. Each sample had 1 ROI that was converted into a data matrix of mass *m*/*z* and signal intensity. Then, orthogonal partial least squares discrimination analysis (OPLS-DA) was performed by EZinfo (Waters, Milford, MA, USA) for the differentiation of samples with different quality characters and for the discovery of the potential quality-associated markers.

## Results

### Quality-Associated Macroscopic and Microscopic Characters of Isatidis Radix

**Figure 1:**
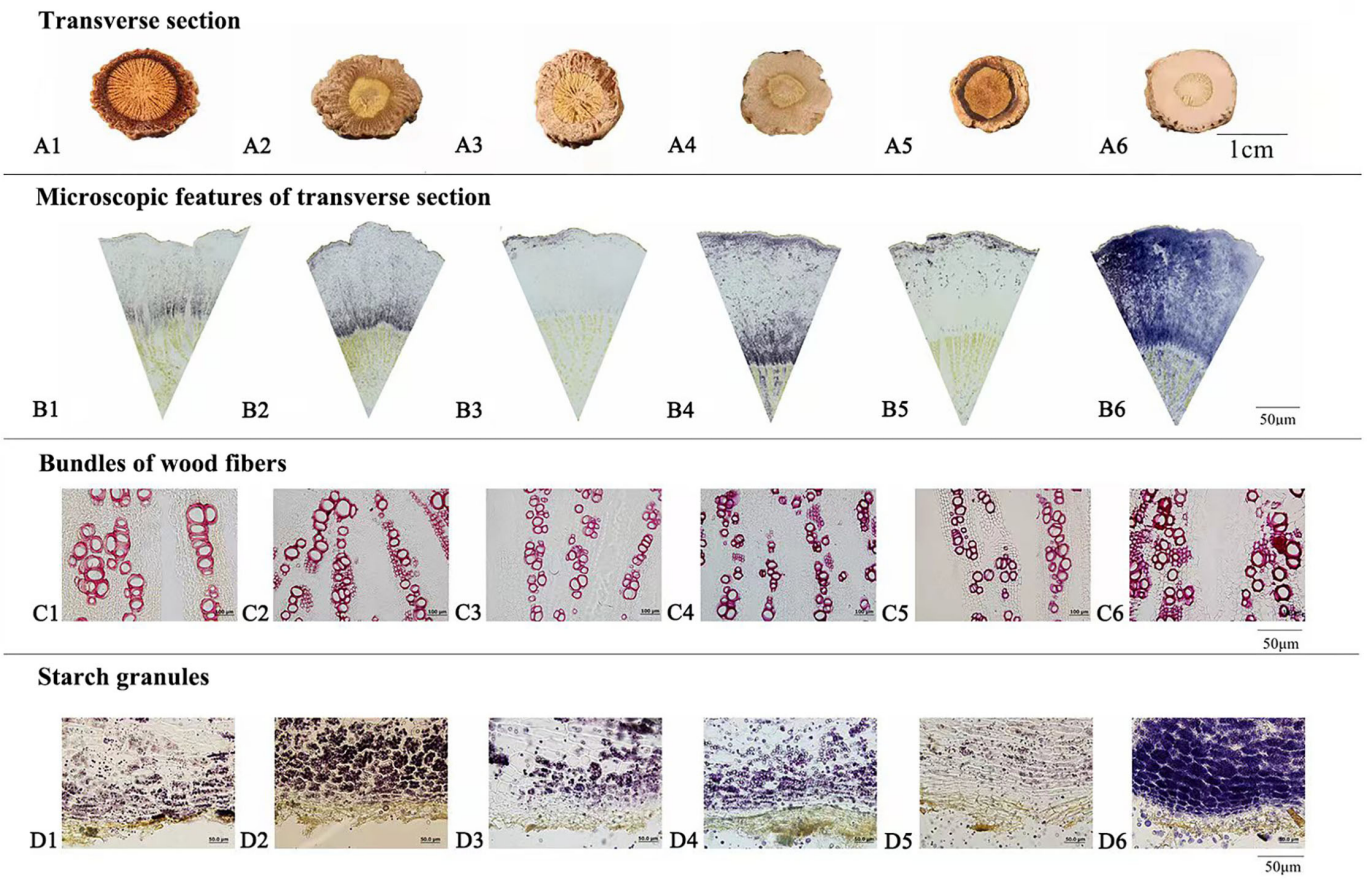
Macroscopic features of transverse section **(A)**, microscopic features of transverse section **(B)**, bundles of wood fibers **(C)**, and starch granules **(D)** of Isatidis Radix with quality character code 1–6.

Macroscopic examination on 64 batches of Isatidis Radix from different markets and habitats was performed. The results showed that the surfaces of major samples were gray, while those of the minor samples were black. Rootheads were observed in most samples except samples S22, S33, and S43. Regarding the lengths and diameters of Isatidis Radix, large spans were measured in different roots from the same batch. Likewise, the depth of longitudinal wrinkle varied without regularity. In contrast to the above features, the texture and characteristics of the transverse section of 64 samples could be categorized into 6 types named with quality character code 1–6 (see Table 2 and Figure 1). To take a closer look at the microscopic features of Isatidis Radix, axial slices of the samples were stained and observed. Normally, the lignified cell wall would be stained red in varying degrees depending on the extent of lignification. On the contrary, starch granules in the cell would be stained blue, and their distribution could be observed clearly. Just as expected, macroscopic and microscopic characteristics of Isatidis Radix were highly related. As shown in Table 2 and Figure 1, when the root was lax and the transverse section was cleft, the bundles of wood fibers and the starch granules were rarely seen under microscopy. Conversely, when the root was compact and the transverse section was oily or starchy, the bundles of wood fibers could be observed easily, and the starch granules were abundant. Nevertheless, exception was found in samples with a quality character code of 5. Although their textures were compact, the transverse sections were horny, and the bundles of wood fibers and the starch granules were scarce, which might be owing to the improper processing and storage of the herbs.

**Table 2:** Quality characters of Isatidis Radix and number of the categorized samples.

**Quality character code**	**Texture**	**Transverse section**	**Bundles of wood fibers**	**Starch granules**	**No. of categorized samples**
1	Lax, soft	With clefts	Rarely visible	Rarely visible	4
2	Compact, slightly soft	Oily	Visible	Abundant	3
3	Lax, hard and brittle	With clefts	Rarely visible	Rarely visible	3
4	Compact, hard and brittle	Oily	Visible	Abundant	38
5	Compact, hard and brittle	Horny	Rarely visible	Rarely visible	4
6	Compact, hard and brittle	Starchy	Visible	Abundant	12

Isatidis Radix has a long history of clinical use and market sale, during which the empirical morphological indicators presenting the superior quality of the herb have been formed. As could be summarized from Table 3, “Isatidis Radix with compact texture and starchy or oily fracture possessing good quality” was the consensus of different classic monographs on herbalogy and pharmacognosy. On the contrary, in the classic monograph “*Species Systematization and Quality Evaluation of Commonly Used Chinese Traditional Drugs* (Northern Edition),” Isatidis Radix is recorded as follows: “The root must be harvested before flowering. If collected after seed bearing, the herb would have a dry surface and a starchy fracture, which is not suitable for medicinal use (Lou, 1995).” Based on the above analysis, samples possessing quality character codes 2, 4, and 6 were considered as superior products. On the contrary, those with quality character codes 1, 3, and 5 were considered as inferior products.

**Table 3:** Quality characters of Isatidis Radix described in classical monographs on herbalogy and pharmacognosy.

**Classical monographs**	**Quality characters**
*Chinese Materia Medica* Chinese Academy of Medical Sciences, 1960	Texture compact and brittle. The herb with obvious starchy fracture was considered a good quality product.
*Handbook of Chinese Materia Medica* Pharmacy Administration, 1959	Texture brittle and easily broken. The herb with oily fracture was considered a good quality product.
*Modern Chinese Materia Medica* Xiao, 1997, *Great Dictionary of Chinese Materia Medica* Nanjing University of Traditional Chinese Medicine., 2015	The herb with compact texture and obvious starchy fracture was considered a good quality product.
*Chinese Drugs* Xu et al., 1993, *Chinese Materia Medica* Editorial Committee of Chinese Materia Medica State Administration of Traditional Chinese Medicine, 1999	The herb with compact texture was considered a good quality product.
*Encyclopedia of Chinese Materia Medica* Cui, 1998	The herb with obvious starchy fracture was considered a good quality product.
*Identification experience of 500 commonly used Chinese Materia Medica* Lu, 1999	The herb with starchy fracture was considered a good quality product.
*Zhong Hua Yao Hai* Ran, 1993	The herb with compact texture and obvious starchy fracture was considered a good quality product.
*Jin shi yuan's traditional identification experience for Chinese Materia Medica* (Jin, 2010)	The herb with oily fracture was considered a good quality product.

### *In situ* Metabolite Profiling in Isatidis Radix by DESI MSI

**Figure 2:**
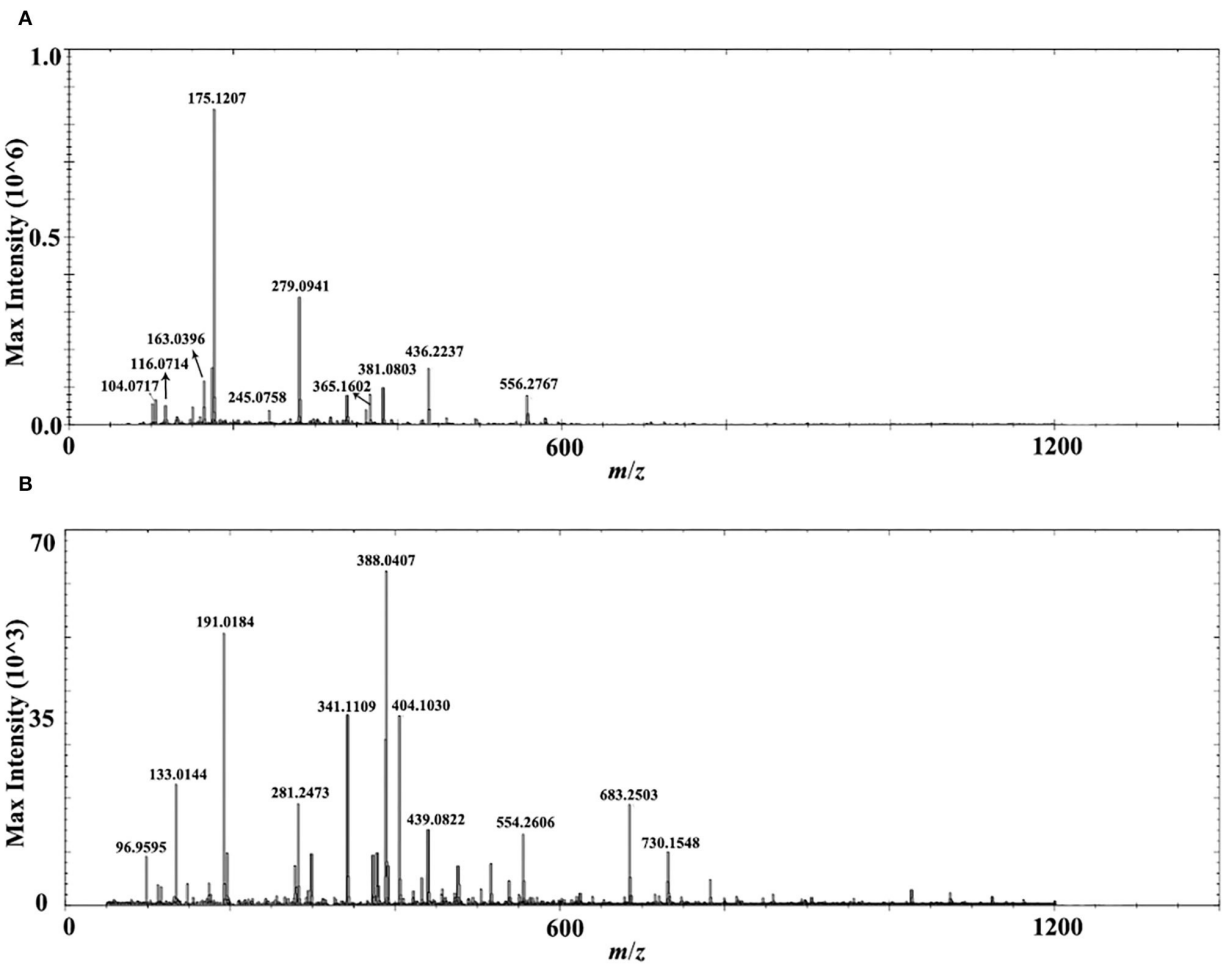
Representative overall average mass spectra acquired from a cross section of Isatidis Radix by desorption electrospray ionization and quadrupole-time-of-flight (DESI-Q-TOF) mass spectrometry imaging (MSI) in the spectral ranges of *m*/*z* 50–1,200 under positive **(A)** and negative **(B)** ionization modes.

A comprehensive metabolite profile of Isatidis Radix was achieved by a high-resolution DESI-Q-TOF/MS. The representative overall average mass spectra under positive and negative ionization modes are shown in Figure 2. The positive ions were mostly detected in the range of *m*/*z* 100–400 and *m*/*z* 550–600, while the negative ions were observed in the range of *m*/*z* 50–800, but with much lower intensity. The putative assignment of the components was based on ions screening using the self-constructed Isatidis Radix UNIFI database and with reference to the isotopic peak, the reference standards, the literature, and other databases. Due to low ion abundances, MS/MS data were difficult to obtain by DESI MSI. Instead, UHPLC-Q-Orbitrap MS/MS analysis was conducted to provide stronger evidence for qualitative identification. As indicated in Supplementary Table 1, a great variety of metabolites including alkaloids, sulfur-containing compounds, phenylpropanoids, nucleosides, amino acids, organic acids, flavonoids, phenols, terpenes, saccharides, peptides, and sphingolipids were detected readily. The positive ions were mainly found as H^+^ adducts of all amino acids, majority of the alkaloids, parts of the phenylpropanoids, the nucleosides and the phenols, several sulfur-containing compounds, a few flavonoids and sphingolipids, as well as organic acids with basic groups. Besides, a small part of alkaloids, phenylpropanoids, nucleosides, phenols, terpenes, peptides, and sulfur-containing compounds were detected in the form of sodium or potassium adduct. At the same time, the negative ions were detected prominently as [M–H]^−^ ions and inconspicuously as [M-H+FA]^−^ ions, evolving the majority of the sulfur-containing compounds and the organic acids, some phenylpropanoids and phenols, a few saccharides, flavonoids, terpenes, as well as alkaloids with acid group. Several components held an isomeric relationship and could not be differentiated by their exact mass, which is listed together in Supplementary Table 1, such as (*R, S*)-goitrin and progoitrin/epiprogoitrin.

### Spatial Distribution of Metabolites in Isatidis Radix by DESI MSI

**Figure 3:**
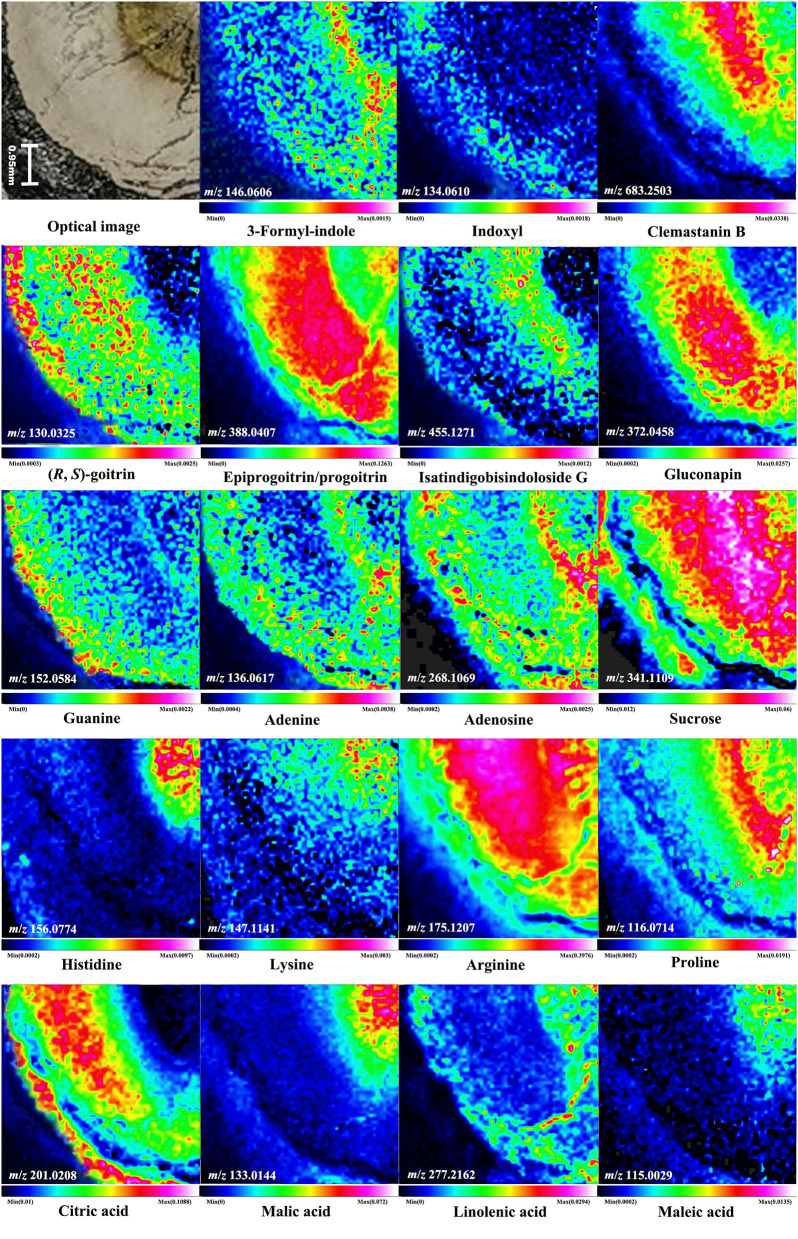
Optical image of Isatidis Radix and the mass spectrometry images of the ions of 3-formyl-indole, indoxyl, clemastanin B, (*R, S*)-goitrin, epiprogoitrin/progoitrin, isatindigobisindoloside G, gluconapin, guanine, adenine, adenosine, sucrose, histidine, lysine, arginine, proline, citric acid, malic acid, linolenic acid, and maleic acid. A color scale from white, pink, red, yellow, green, blue to blank indicates a descending absence of the signals.

The optical image of the transverse section (Figure 3) showed the anatomical structure of Isatidis Radix: xylem, cambium, phloem, cortex, and cork from inside to outside. Thanks to the MSI technology, tissue distributions of different kinds of metabolites could be visualized in these structures. For example, Figure 3 represented the H^+^ adducts of 3-formyl-indole and indoxyl. Although they are both typical alkaloids from Isatidis Radix, a minor difference was observed in their distribution pattern. The former was rich in the cambium and the latter was more abundant in the outer area of phloem. Likewise, the accumulation sites of amino acids and organic acids were also diversified. The signals of [M+H]^+^ ions of histidine and lysine were more obvious in the xylem (Figure 3), while that of proline tended to disperse from cambium to both sides (Figure 3). At the same time, the protonated adduct of arginine, the most abundant amino acid in Isatidis Radix, was located with strong signal intensity in all regions except the cortex and cork (Figure 3). A similar localization pattern (Figure 3) was found for sucrose, another component with high concentration in the herb. Unlike amino acids, organic acids in Isatidis Radix were detected mostly in the form of [M-H]^−^ ions. The accumulation sites of malic acid and maleic acid bore some similarities, which were observed mainly in the xylem (Figure 3). Conversely, the high signal intensity of citric acid was detected in the outer areas of the tissue including phloem, cortex, and cork (Figure 3). Interestingly, the ion of linolenic acid was located in xylem, cortex, and cork, and in the innermost and outermost regions of the root (Figure 3).

The (*R, S*)-goitrin, the mixture of epigoitrin [(*R*)-goitrin] and goitrin [(*S*)-goitrin-] (Nie et al., 2016, 2017), was one of the most characteristic sulfur-containing compounds in Isatidis Radix and was acknowledged as the quality marker of this herb (Chinese Pharmacopoeia Commission, 2020). Epigoitrin and goitrin were verified as the breakdown products of their glucosinolate prototypes, epiprogoitrin and progoitrin, which also existed in Isatidis Radix (Nie et al., 2011). Epigoitrin, goitrin, epiprogoitrin, and progoitrin exhibited anti-influenza activity *in vitro*/*ovo* (Nie et al., 2020), and they were detected in the cross-section of Isatidis Radix by DESI MSI. As indicated in Figure 3, positive ion of (*R, S*)-goitrin and negative ion of epiprogoitrin/progoitrin all showed dominant abundance in the phloem. When investigated further, different preferential occurrences of (*R, S*)-goitrin and epiprogoitrin/progoitrin were observed in the cortex, cork, and the inner core of the xylem, respectively. Similar to epiprogoitrin/progoitrin, isatindigobisindoloside G and gluconapin, another two glucosinolates in Isatidis Radix, were mainly detected as the [M-H]^−^ ions that accumulated mostly in phloem (Figure 3). Conversely, the negative ion of clemastanin B, an impotent anti-influenza phenylpropanoid discovered from Isatidis Radix (Yang et al., 2013), presented the highest abundance in the cambium and around (Figure 3). Apart from the anti-viral effect, Isatidis Radix posed anti-inflammatory function as well. According to the literature, the major therapeutic reagents were nucleosides including guanine, adenine, and adenosine (Evaldsson et al., 2007; Clercq, 2019; Wu et al., 2019). As noticeable in Figure 3, the proton adducts of the above-mentioned nucleosides were located almost in the cambium and the outer area of the phloem. In particular, adenosine and its nucleobase, adenine, shared nearly the same tissue distribution.

### Potential Quality-Associated Markers for Isatidis Radix Discovered by DESI MSI Combined With OPLS-DA

**Figure 4:**
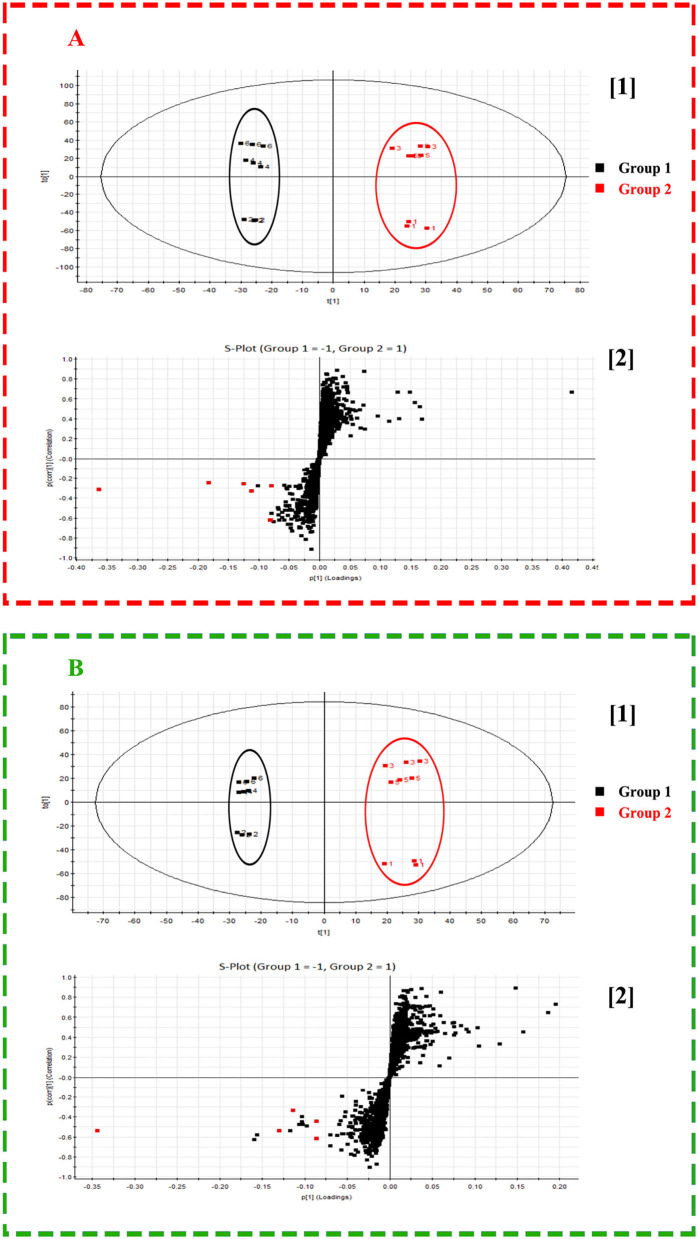
Score plot ([1]) and S-plot ([2]) obtained from orthogonal partial least squares discrimination analysis (OPLS-DA) results of Radix Isatidis with quality character code 1–6 based on desorption electrospray ionization and quadrupole-time-of-flight (DESI-Q-TOF) mass spectrometry imaging (MSI) in the spectral ranges of *m*/*z* 50–1,200 under positive **(A)** and negative **(B)** ionization modes. The potential quality-associated markers for Isatidis Radix were tagged to red dots.

To explore the potential of mass spectrometry imaging in quality illustration of herbal medicine, every 3 batches of representative Isatidis Radix possessing 6 categories of quality characters (indicated in Table 2) were analyzed by DESI-Q-TOF/MS. Although the metabolites' spatial distribution pattern bore some similarities in different categories of samples, their relative signal intensity in smaller tissues varied. The pattern recognition method OPLS-DA can distinguish among different groups and, simultaneously, identify the major contributing components. In our cases, OPLS-DA models based on MSI data of the cross-section within the whole spectral ranges (*m*/*z* 50–1,200) under positive and negative modes were further established to clarify the difference between superior and inferior samples. As analyzed in the “Quality-associated macroscopic and microscopic characters of Isatidis Radix” section, respective 3 batches of Isatidis Radix with quality characters codes 2, 4, and 6 were classified in group 1, representing good quality. Conversely, respective 3 batches of Isatidis Radix with quality character codes 1, 3, and 5 were classified in group 2, representing poor quality. As shown in Figures 4A[1],B[1], these two groups could be clearly clustered in the score plots of both positive and negative ion modes. Furthermore, the major differential components were acquired from the S-plots (Figures 4A[2],B[2]), in which each point represented an ion. The farther a data point was from the center point, the greater that ion contributed to the sample difference. Normally, compounds distributed at the two ends of the S-plot were regarded as the potential markers. Among them, arginine, progoitrin/epiprogoitrin, (*R, S*)-goitrin, 3-formyl-indole, syringin, adenosine, malic acid, adenine, isovitexin, clemastanin B, and uridine exerted higher ion intensities in samples from group 1 than in those from group 2. The very important in projection (VIP) values for their corresponding ions were 17.90, 18.77, 8.93, 7.08, 6.31, 6.10, 5.99, 5.50, 4.35, 4.31, and 4.08, respectively, with *P*-values all below 0.05. In all, these components could be used as potential quality-associated markers for Isatidis Radix.

## Discussion

Compared with MALDI, DESI has the advantages of minimal sample preparation and less requirements for tissue sectioning. Nevertheless, the dry, hard, and fragile texture of Isatidis Radix still brought great challenges. First, the thickness of the slice was optimized from 40 μm to 0.5 cm. The scanning results indicated that although thick slices above 0.1 cm could ensure tissue integration and could be obtained easily by an ordinary knife or blade, their ion intensity was rather poor. On the contrary, notwithstanding thin slices below 100 μm acquired from cryosectioning afforded satisfactory detection sensitivity, they tended to be blown away from the glass slide by the spray gas of DESI, even adhered by various ways. Taking a comprehensive consideration of the quality of the mass data and the integrity of the tissue, a thickness of 100 μm was selected. In addition, fixing methods of the slice were optimized to prevent blowing. It was found that when the tissue was thaw-mounted directly on the glass slide with or without pretreatment of the 0.1% (w/v) poly(*L*-lysine) hydrobromide solution, they could be easily blown away. When investigated further, the double-sided adhesive tape worked better than other tapes. So, in our case, a 100-μm-thick slice of Isatidis Radix was cryosectioned and fixed on a glass slide with the double-sided conductive tape.

After the optimization of the preprocess method, ions of alkaloids, sulfur-containing compounds, phenylpropanoids, nucleosides, amino acids, organic acids, flavonoids, phenols, terpenes, saccharides, peptides, and sphingolipids were detected by DESI MSI in the cross-section of the root without extraction and isolation. In comparison with our previous results obtained from MALDI MSI (Nie et al., 2021), the signal sensitivity of DESI under negative mode was lower. But DESI performed better for nucleosides and (*R, S*)-goitrin, the assay marker of Isatidis Radix in Chinese Pharmacopeia, edition 2020 (Chinese Pharmacopoeia Commission, 2020). Although the spatial resolution of DESI was lower due to the ionization principle involving spray, it had the advantages of simpler sample preparation and small molecule applicability.

**Figure 5:**
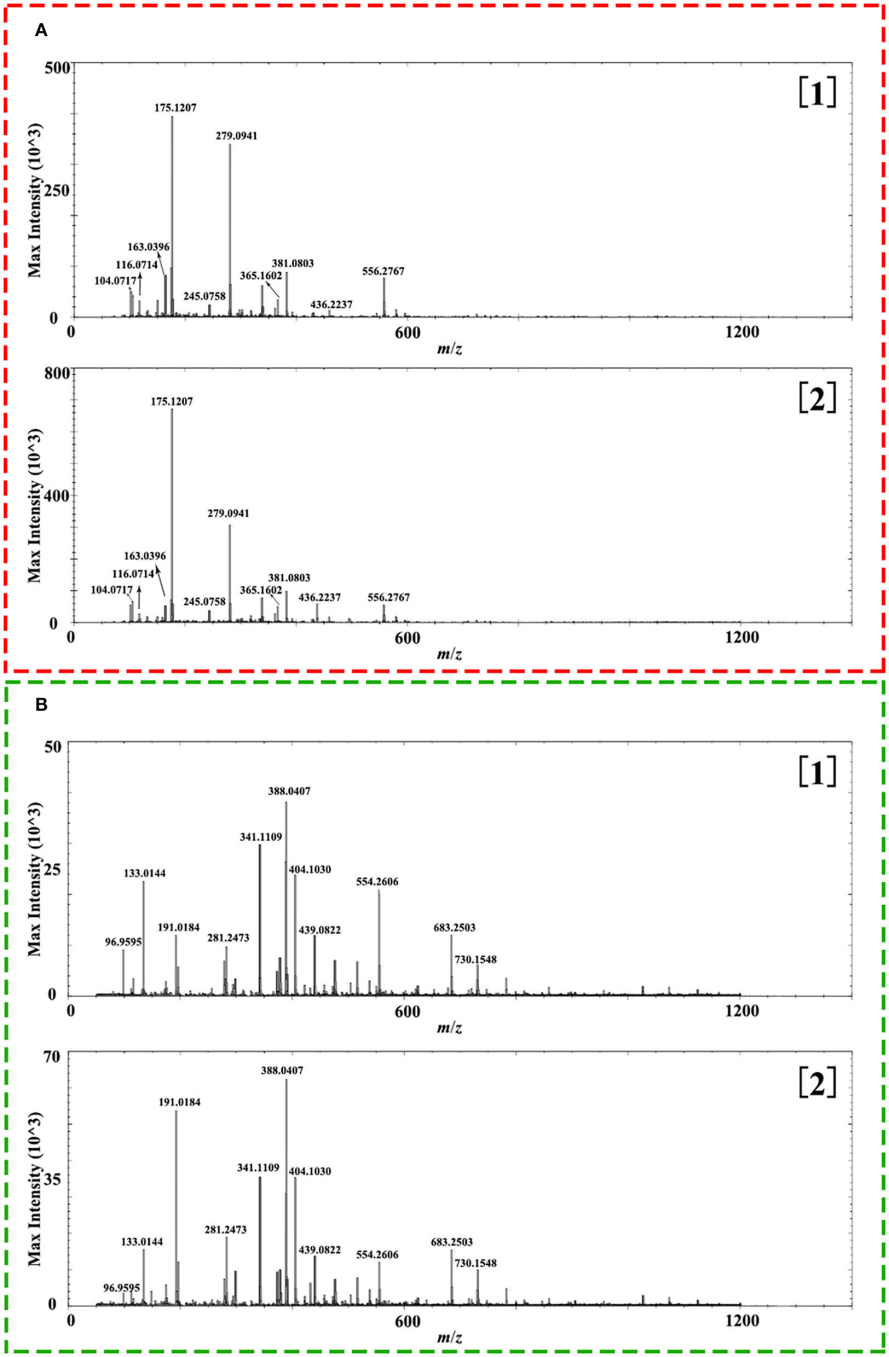
Representative overall average mass spectra acquired from wood ([1]) and bark ([2]) of the cross section of Isatidis Radix by desorption electrospray ionization and quadrupole-time-of-flight (DESI-Q-TOF) mass spectrometry imaging (MSI) in the spectral ranges of *m*/*z* 50–1,200 under positive **(A)** and negative **(B)** ionization modes.

As presented in Figure 3, the transverse section of Isatidis Radix could be roughly divided into 2 parts, namely, bark (including cork, cortex, and phloem) and wood (including cambium and xylem). As indicated in Figure 5, metabolites showed higher ion intensity in the bark than in the wood. When the proportions of the wood region in the whole transverse section were measured and calculated, it was interesting to find that superior and inferior Isatidis Radix show distinct results. The wood of samples with quality character codes 2, 4, and 6 occupied approximately 1/3–1/2 of the whole transverse section, whereas that of samples with quality character codes 1, 3, and 5 occupied approximately 3/5–4/5. In brief, herbs with poorer quality indicated higher proportion of wood and less chemical components. The consistency proved the power of MSI to illustrate the quality-related morphological features of herbal medicine.

Until now, 18 classes of metabolites have been isolated from Isatidis Radix, including alkaloids, sulfur-containing compounds, phenylpropanoids, amino acids, nucleosides, organic acids and esters, flavonoids, quinones, terpenes, sterols, saccharides, aromatics, peptides, alcohols, aldehydes and ketones, nitriles, and sphingolipids (Lin et al., 2005; Speranza et al., 2020). On the contrary, it is commonly used in clinic for treatment and prohibition against fever and infection (Zhou and Zhang, 2013). In contrast with its diversified composition and accurate clinical efficacy, the quality control of this herbal medicine is still insufficient. One of the reasons might be that individual chemical markers failed to show satisfactory pharmacological effects as the herb did. This work explored a novel way to reveal the quality indicators of Isatidis Radix. By employing the MSI as a bridge, the morphological indicators presenting superior quality based on the long history of clinical use and market sale were illustrated from a spatial chemistry perspective. Combining with chemometric analysis, the potential quality markers were discovered ultimately.

## Conclusion

In this work, desorption electrospray ionization (DESI) mass spectrometry imaging (MSI) was unprecedentedly performed to unravel the distribution of metabolites in the cross-section of Isatidis Radix and distinguish samples with 6 different quality characters. To ensure adequate detection sensitivity and prevent blown off of the slice, section and fixing methods of the tissue were optimized. In all, 102 components were assigned, and spatial contexts of some components in the root tissue were mapped. Combined with OPLS-DA, superior and inferior herbs were distinguished unambiguously in accordance with their mass spectrometry images. Ultimately, markers relating to the quality characters of Isatidis Radix were discovered, which exerted stronger spatial signals in samples with good quality and showed significant influence on differentiation. Pharmacological research of the markers and determination of their contents in Isatidis Radix are underway in our laboratory. Above all, the novel method was useful for characterizing metabolomes in histological tissues, linking the chemicals and morphological features, thus providing the scientific basis for quality evaluation of herbal medicine.

## Data Availability Statement

The original contributions presented in the study are included in the article/Supplementary Material, further inquiries can be directed to the corresponding author/s.

## Author Contributions

L-XN and L-YH designed and performed the experiments, analyzed the data, and wrote the manuscript. X-PW, L-FL, X-XY, and X-FJ assisted in performing the experiments. SK collected and authenticated the samples. L-WY, ZD, and S-CM revised the manuscript. All authors read and approved the final manuscript.

## Funding

This study was supported by the National Natural Science Foundation of China (Grant No. 81303194).

## Conflict of Interest

X-XY and X-FJ are employed by Waters Corporation. The remaining authors declare that the research was conducted in the absence of any commercial or financial relationships that could be construed as a potential conflict of interest.

## Publisher's Note

All claims expressed in this article are solely those of the authors and do not necessarily represent those of their affiliated organizations, or those of the publisher, the editors and the reviewers. Any product that may be evaluated in this article, or claim that may be made by its manufacturer, is not guaranteed or endorsed by the publisher.
