# Supplementary material for: Desorption Electrospray Ionization Mass Spectrometry Imaging Illustrates the Quality Characters of Isatidis Radix
Source: Front Plant Sci. 2022 Jun 15;13:897528. doi: 10.3389/fpls.2022.897528 (PMC9240750; doi:10.3389/fpls.2022.897528)
Supplement: Supplementary file 1 [file Table_1.docx]

# Supplementary Table 1 Metabolites assigned in cross-section of Isatidis Radix by desorption electrospray ionization combined with quadrupole-time-of-flight mass spectrometry (DESI-Q-TOF) mass spectrometry imaging (MSI)

| **No** | **Compound** | **Chemical class** | **Ion formula** | **Theoretical *m*/*z*** | **Observed *m*/*z*** | **Mass accuracy (ppm)** | **Fragments**  ***m*/*z*** | **Ref.** |
| --- | --- | --- | --- | --- | --- | --- | --- | --- |
| 1 | Alanine | Amino acids | C_3_H_7_NO_2_+H | 90.0556 | 90.0559 | 3.3 | / | Pan, 2014 |
| 2 | *r*-Aminobutyric acid | Amino acids | C_4_H_9_NO_2_+H | 104.0712 | 104.0717 | 4.8 | 87.0441 | Pan, 2014 |
| 3 | Choline | Alkaloids | C₅HON⁺ | 104.1075 | 104.1069 | 5.8 | / | The Metabolomics Innovation Centre, 2020 |
| 4 | Uracil | Nucleosides | C_4_H_4_N_2_O_2_+H | 113.0345 | 113.0350 | 4.4 | 96.0088, 70.0296 | Pan, 2013 |
| 5 | Proline | Amino acids | C_5_H_9_NO_2_+H | 116.0712 | 116.0714 | 1.7 | 70.0651 | Wu et al., 1997 |
| 6 | Valine | Amino acids | C_5_H_11_NO_2_+H | 118.0869 | 118.0867 | 1.7 | 72.0816 | Chen et al., 2012 |
| 7 | (*R*, *S*)-goitrin | Sulphur-containing compounds | C_5_H_7_NOS+H | 130.0327 | 130.0325 | 1.5 | 96.0455, 70.0663 | Wang et al., 2014 |
| 8 | Indoxyl | Alkaloids | C_8_H_7_NO+H | 134.0600 | 134.0610 | 7.5 | 124.0542, 116.0500 | Maugard et al., 2001 |
| 9 | 6-Hydroxyindole | Alkaloids | C_8_H_7_NO+H | 134.0600 | 134.0610 | 7.5 | 116.0453 | Waters, 2020 |
| 10 | Adenine | Nucleosides | C_5_H_5_N_5_+H | 136.0624 | 136.0617 | 5.1 | 119.0357, 94.9688 | Chen et al., 2012 |
| 11 | Aminobenzoic acid | Organic acids | C_7_H_7_NO_2_+H | 138.0556 | 138.0550 | 4.3 | 120.0393 | Wang et al., 2013 |
| 12 | 3-Formyl-indole | Alkaloids | C_9_H_7_N_1_O+H | 146.0607 | 146.0606 | 0.7 | 118.0653 | Zhao, 2015 |
| 13 | 4(3*H*)-quinazolinone | Alkaloids | C_8_H_6_N_2_O+H | 147.0480 | 147.0471 | 6.1 | 129.0360 | Liu et al., 2018 |
| 14 | Lysine | Amino acids | C_6_H_14_N_2_O_2_+H | 147.1134 | 147.1141 | 4.8 | 84.0821 | Pan, 2014 |
| 15 | Guanine | Nucleosides | C_5_H_5_N_5_O+H | 152.0573 | 152.0584 | 7.2 | / | Pan, 2013 |
| 16 | Histidine | Amino acids | C_6_H_9_N_3_O_2_+H | 156.0774 | 156.0774 | 0.0 | 110.0715 | Pan, 2014 |
| 17 | Arginine | Amino acids | C_6_H_14_N_4_O_2_+H | 175.1206 | 175.1207 | 0.6 | 158.0932 | Zeng et al., 2010 |
| 18 | 1-Methoxy-3-indolecar-baldehyde | Alkaloids | C_10_H_9_NO_2_+H | 176.0706 | 176.0713 | 4.0 | 158.0607 | Waters, 2020 |
| 20 | Tyrosine | Amino acids | C_9_H_11_NO_3_+H | 182.0818 | 182.0836 | 9.9 | 165.0557 | Pan, 2014 |
| 20 | Dihydroconiferyl alcohol | Phenylpropanoids | C_10_H_14_O_3_+H | 183.1022 | 183.1014 | 4.4 | / | Wang et al., 2013 |
| 21 | *L*-Targinine | Peptides | C_7_H_16_N_4_O_2_+Na | 211.1165 | 211.1180 | 7.1 | 172.0905 | The Metabolomics Innovation Centre, 2020 |
| 22 | (1'*R*, 2'*R*, 3'*S*, 4'*R*)-1, 2, 4-triazole | Nucleosides | C_7_H_11_N_3_O_4_+K | 240.1733 | 240.1724 | 3.7 | / | Pan, 2014 |
| 23 | Cytidine | Nucleosides | C_9_H_13_N_3_O_5_+H | 244.0934 | 244.0938 | 1.6 | 112.0504 | Zhao, 2015 |
| 24 | Uridine | Nucleosides | C_10_H_13_N_5_O_4_+H | 245.0774 | 245.0758 | 6.5 | 113.0349 | Zhao, 2015 |
| 25 | Vomifoliol | Terpenes | C_13_H_20_O_3_+Na | 247.1310 | 247.1320 | 3.6 | / | Wang et al., 2013 |
| 26 | 2-Pyrrolidone-*N*-methylene-*β*-D-arabinoside | Alkaloids | C_10_H_17_NO_6_+H | 248.1135 | 248.1135 | 0.0 | / | Pan, 2014 |
| 27 | (*S*)-(-)-spirobrassinin | Sulphur-containing compounds | C_11_H_10_N_2_OS_2_+H | 251.0314 | 251.0322 | 3.2 | / | Zhang et al., 2020b |
| 28 | 2'-Deoxyadenosine | Nucleosides | C_10_H_13_N_5_O_3_+H | 252.1097 | 252.1096 | 0.4 | 120.0672 | Pan, 2014 |
| 29 | Pyrraline | Amino acids | C_12_H_18_N_2_O_4_+H | 255.1339 | 255.1358 | 7.4 | / | The Metabolomics Innovation Centre, 2020 |
| 30 | Hexadecanamide | Alkaloids | C_16_H_33_NO+H | 256.2641 | 256.2641 | 0.0 | 102.0923, 88.0763 | China Pharmaceutical University, 2020 |
| 31 | Liquiritigenin | Flavonoids | C_15_H_12_O_4_+H | 257.0808 | 257.0797 | 4.3 | 239.0692 | Peng et al., 2005a |
| 32 | Indirubin/Indigotin | Alkaloids | C_16_H_10_N_2_O_2_+H | 263.0813 | 263.0827 | 5.3 | 245.0716, 235.0866 | Chen et al., 2012 |
| 33 | Adenosine | Nucleosides | C_10_H_13_N_5_O_4_+H | 268.1047 | 268.1069 | 8.2 | 250.1075 | Zhao, 2015 |
| 34 | Inosine | Nucleosides | C_10_H_12_N_4_O_5_+H | 269.0887 | 269.0900 | 4.8 | 137.0456 | Pan, 2013 |
| 35 | 2'-Deoxyinosine | Nucleosides | C_10_H_12_N_4_O_4_+Na | 275.0757 | 275.0740 | 6.2 | 143.0331 | Pan, 2014 |
| 36 | 2′-*O*-methyladenosine | Nucleosides | C_11_H_15_N_5_O_4_+H | 282.1203 | 282.1200 | 4.6 | 147.0656, 136.0621 | Zhang et al., 2019a |
| 37 | Indican | Alkaloids | C_14_H_17_NO_6_+H | 296.0925 | 296.0938 | 4.4 | 278.0815 | Zou and Koh., 2007 |
| 38 | Guanosine | Nucleosides | C_10_H_13_N_5_O_5_+Na | 306.0815 | 306.0798 | 5.6 | 152.0577 | Xiao et al., 2007 |
| 39 | Phaitanthrin A/Epiphaitanthrin A | Alkaloids | C_18_H_14_N_2_O_3_+H | 307.1083 | 307.1063 | 6.5 | / | Liu et al., 2016 |
| 40 | Cephalanthrin A | Alkaloids | C_17_H_12_N_2_O_4_+H | 309.0876 | 309.0852 | 7.8 | / | Zhang et al., 2020b |
| 41 | Indiforine F | Alkaloids | C_14_H_18_N_2_O_5_+Na | 317.1114 | 317.1129 | 4.7 | / | Liu et al., 2018 |
| 42 | Evofolin-B | Phenylpropanoids | C_17_H_18_O_6_+H | 320.1182 | 320.1208 | 8.1 | / | Wang et al., 2013 |
| 43 | Indole-3-acetonitrile-6-*O*-*β*-D-glucopyranoside | Alkaloids | C_16_H_18_N_2_O_6_+H | 335.1244 | 335.1229 | 4.5 | 187.0330, 160.0211 | He et al., 2006a |
| 44 | Isatisindigoticanine K | Alkaloids | C_19_H_13_N_3_O_2_+Na | 338.0906 | 338.0899 | 2.1 | / | Zhang et al., 2020c |
| 45 | Dihydroneoascorbigen | Alkaloids | C_16_H_19_NO_7_+H | 338.1299 | 338.1271 | 8.3 | / | Zhang et al., 2020b |
| 46 | Erucamide | Alkaloids | C_22_H_43_NO+H | 338.3424 | 338.3441 | 5.0 | / | China Pharmaceutical University, 2020 |
| 47 | (-)-(*R*)-2-(3-Cyanomethyl-4-methoxy-1*H*-indol-7-yl)-2-(1*H*-indol-3-yl)acetonitrile | Alkaloids | C_22_H_18_N_4_O_2_+H | 341.1403 | 341.1417 | 4.1 | 291.1021, 223.0625 | Chen et al., 2012 |
| 48 | Isatiscaloids B | Alkaloids | C_16_H_20_N_2_O_5_+Na | 343.1270 | 343.1256 | 4.1 | 240.0331, 217.0963 | Wang et al., 2013 |
| 49 | Coniferin | Phenylpropanoids | C_16_H_22_O_8_+H | 343.1394 | 343.1376 | 5.2 | 181.0863 | Zhang et al., 2019a |
| 50 | (-)-Salutaridine | Alkaloids | C_19_H_21_NO_4_+K | 366.1103 | 366.1094 | 2.5 | / | Waters, 2020 |
| 51 | Indole-3-acetonitrile-2-*S*-*β*-D-glucopyranoside | Sulphur-containing compounds | C_16_H_18_N_2_O_5_S+Na | 373.0834 | 373.0866 | 8.6 | 189.0331 | Yang et al., 2014b |
| 52 | Syringin | Phenylpropanoids | C_17_H_24_O_9_+Na | 395.1318 | 395.1353 | 8.9 | 193.0844, 133.0686 | Zhang et al., 2019a |
| 53 | (-)-5-Methoxyisolariciresinol | Phenylpropanoids | C_21_H_26_O_7_+Na | 413.1577 | 413.1588 | 2.7 | / | Wang et al., 2013 |
| 54 | Isatindigobisindoloside G | Sulphur-containing compounds | C_23_H_22_N_2_O_6_S+H | 455.1278 | 455.1271 | 1.5 | 293.0742 | Zhang et al., 2020a |
| 55 | Isatindigobisindoloside E | Sulphur-containing compounds | C_23_H_24_N_2_O_7_+Na | 463.1482 | 463.1464 | 3.9 | 279.0951 | Liu et al., 2015a |
| 56 | Isatigotindolediosides A | Alkaloids | C_21_H_27_NO_12_+H | 486.1612 | 486.1627 | 3.1 | 468.1501 | Meng et al., 2017b |
| 57 | Isatithioetherin A/Isatithioetherin B | Sulphur-containing compounds | C_20_H_26_N_4_O_4_S_3_+Na | 505.1014 | 505.1016 | 0.4 | / | Meng et al., 2017b |
| 58 | Bisindigotin | Alkaloids | C_32_H_18_N_4_O_2_+Na | 513.1328 | 513.1355 | 5.3 | 473.1217 | Wei et al., 2005 |
| 59 | Isatithioetherin C/Isatithioetherin E | Sulphur-containing compounds | C_20_H_26_N_4_O_4_S_4_+H | 515.0916 | 515.0939 | 4.5 | / | Meng et al., 2017b |
| 60 | Isatindigoside E | Alkaloids | C_30_H_25_N_3_O_7_+H | 540.1772 | 540.1757 | 2.8 | 522.1610 | Zhang et al., 2020b |
| 61 | (+)-(7*R*, 7'*R*, 8*S*, 8'*S*)-neo-olivil | Phenylpropanoids | C_26_H_34_O_12_+Na | 561.2048 | 561.2038 | 1.8 | / | Kikuchi et al., 2005 |
| 62 | *E*-2-[(3'-indole)cyanomethylene]-3-indolinone | Alkaloids | C_18_H_11_N_3_O*2+H | 601.2346 | 601.2307 | 6.5 | / | Waters, 2020 |
| 63 | Isoscoparin-3'-*O*-glucopyranoside | Flavonoids | C_28_H_34_O_16_+K | 649.1165 | 649.1179 | 2.2 | 487.0631 | Waters, 2020 |
| 64 | 1-*O*-*β*-D-glucopyranosyl-(2*S*, 3*R*)-*N*-(2'-hydroxyhe xacosanoyl)-octadeca-11*E*-sphingenine | Sphingolipids | C_50_H_97_NO_9_+H | 856.7242 | 856.7308 | 7.7 | / | Sun et al., 2009 |
| 65 | Propanedioic acid | Organic acids | C_3_H_4_O_4_-H | 103.0031 | 103.0022 | 8.7 | 84.9921 | Li, 2010 |
| 66 | Maleic acid | Organic acids | C_4_H_4_O_4_-H | 115.0031 | 115.0029 | 1.7 | 71.0123 | Peng et al., 2005b |
| 67 | Succinic acid | Organic acids | C_4_H_6_O_4_-H | 117.0203 | 117.0186 | 6.0 | 99.0090 | Sun et al., 2007 |
| 68 | Malic acid | Organic acids | C_4_H_6_O_5_-H | 0136 | 133.0144 | 6.0 | 115.0035, 89.0233 | Liu et al., 2010 |
| 69 | 2-Amino benzoic acid | Organic acids | C_7_H_7_NO_2_-H | 136.0398 | 136.0397 | 0.7 | 18.0286 | Li, 2010 |
| 70 | Salicylic acid | Organic acids | C_7_H_6_O_3_-H | 137.0238 | 137.0239 | 0.7 | 93.0339 | Zhao, 2015 |
| 71 | Fructose/Glucose | Saccharides | C_6_H_12_O_6_-H | 179.0555 | 179.0559 | 2.2 | 119.0330 | Liu et al., 2010 |
| 72 | Citric acid | Organic acids | C_6_H_8_O_7_-H | 201.0201 | 201.0208 | 8.9 | 183.0097 | Liu et al., 2010 |
| 73 | Glucuronic acid | Organic acids | C_6_H_10_O-H | 203.0348 | 203.0331 | 8.8 | 185.0236 | Liu et al., 2010 |
| 74 | Isatindosulfonic acid C | Sulphur-containing compounds | C_10_H_11_NO_4_S-H | 240.0330 | 240.0345 | 6.2 | / | Meng et al., 2017a |
| 75 | Palmitic acid | Organic acids | C_16_H_32_O_2_-H | 255.2323 | 255.2339 | 6.3 | 256.2408 | Kizil et al., 2009 |
| 76 | 3-[2'-(5'-Hydroxymethyl)furyl]-1(2*H*)-isoquinolinone-7-*O*-*β*-D-glucoside | Alkaloids | C_20_H_21_NO_9_-H | 256.0615 | 256.0627 | 4.7 | 94.0081 | He et al., 2006b |
| 77 | Linolenic acid | Organic acids | C_18_H_30_O_2_-H | 277.2167 | 277.2162 | 1.8 | 241.1955, 121.1011 | Kizil et al., 2009 |
| 78 | Stearic acid | Organic acids | C_18_H_36_O_2_-H | 283.2636 | 283.2652 | 5.6 | 265.2535 | Kizil et al., 2009 |
| 79 | Tryptanthrin | Alkaloids | C_15_H_8_N_2_O_2_-H+FA | 293.0565 | 293.0583 | 6.1 | / | Chen et al., 2012 |
| 80 | Isaindigodione | Alkaloids | C_18_H_18_N_2_O_4_-H | 325.1204 | 325.1186 | 2.5 | / | Xiao et al., 2007 |
| 81 | Sucrose | Saccharides | C_12_H_22_O_11_-H | 341.1083 | 341.1109 | 7.6 | 179.0567, 161.0454, 89.0233 | Liu et al., 2010 |
| 82 | Adenosine monophosphate | Nucleosides | C_10_H_14_N_5_O_7_P-H | 346.0552 | 346.0563 | 3.2 | 268.1065 | Guo et al., 2020 |
| 83 | Sinigrin | Sulphur-containing compounds | C_10_H_17_NO_9_S_2_-H | 358.0266 | 358.0276 | 2.8 | 116.0167 | Guo et al., 2020 |
| 84 | Lariciresinol/(+)-Isolariciresinol | Phenylpropanoids | C_20_H_24_O_6_-H | 359.1494 | 359.1469 | 7.0 | 329.1401, 192.0788 | Li et al., 2010 |
| 85 | Sinensetin | Flavonoids | C_20_H_20_O_7_-H | 371.1130 | 371.1126 | 1.1 | 353.1028 | Li, 2010 |
| 86 | Gluconapin | Sulphur-containing compounds | C_11_H_19_NO_9_S_2_-H | 372.0422 | 372.0458 | 9.7 | 130.0324 | Mohn et al., 2007 |
| 87 | Isatindigotindoloside C/Isatindigotindoloside D | Sulphur-containing compounds | C_17_H_20_N_2_O_6_S-H | 379.0963 | 379.0978 | 4.0 | 137.0876 | Liu et al., 2015b |
| 88 | Corchoionoside C | Terpenes | C_19_H_30_O_8_-H | 385.1862 | 385.1883 | 5.5 | / | Zhang et al., 2019a |
| 89 | Progoitrin/Epiprogoitrin | Sulphur-containing compounds | C_11_H_19_NO_10_S_2_-H | 388.0371 | 388.0407 | 9.3 | 128.0247 | Mohn et al., 2007 |
| 90 | Gluconapoleiferin | Sulphur-containing compounds | C_12_H_21_NO_10_S_2_-H | 402.0858 | 402.0864 | 1.5 | / | Peng et al., 2005b |
| 91 | Isatindigoside H | Alkaloids | C_23_H_24_N_2_O_6_-H | 423.1555 | 423.1541 | 3.3 | 261.1023 | Zhang et al., 2020c |
| 92 | Isatindigobisindoloside F | Alkaloids | C_23_H_22_N_2_O_7_-H | 437.1348 | 437.1337 | 2.5 | 275.0823 | Liu et al., 2015a |
| 93 | Glucobrassicin | Sulphur-containing compounds | C_16_H_20_N_2_O_9_S_2_-H | 447.0531 | 447.0562 | 6.9 | 205.0431 | Guo et al., 2020 |
| 94 | Neoglucobrassicin | Sulphur-containing compounds | C_17_H_22_N_2_O_10_S_2_-H | 477.0637 | 477.0621 | 3.4 | 234.9657 | Guo et al., 2020 |
| 95 | Isatindigoside J | Alkaloids | C_25_H_27_N_3_O_8_-H | 496.1720 | 496.1755 | 7.3 | 334.1188 | Zhang et al., 2020c |
| 96 | Isatigotindolediosides F | Sulphur-containing compounds | C_21_H_27_NO_12_S-H | 516.1175 | 516.1139 | 7.0 | 274.1083 | Meng et al., 2017b |
| 97 | Linarin | Flavonoids | C_28_H_32_O_14_-H | 591.1713 | 591.1753 | 6.8 | 429.1182 | Peng et al., 2005a |
| 98 | Neohesperidin | Flavonoids | C_28_H_34_O_15_-H | 609.1820 | 609.1788 | 5.1 | 447.1286 | Peng et al., 2005a |
| 99 | Isovitexin-3'-*O*-glucopyranoside/Isovitexin-6'-*O*-glucopyranoside | Flavonoids | C_26_H_28_O_15_-H+FA | 625.1410 | 625.1415 | 0.8 | 341.0670 | Waters, 2020 |
| 100 | Clemastanin B | Phenylpropanoids | C_32_H_44_O_16_-H | 683.2550 | 683.2503 | 6.9 | 521.2026, 359.1493 | Yang et al., 2014a |
| 101 | Isovitexin | Flavonoids | C_21_H_20_O_10_*2-H | 835.1727 | 835.1770 | 5.1 | 341.0673, 283.0614 | Zhao, 2015 |
| 102 | Indigoticoside A/Lariciresinol-4'-*O*-*β*-D-glucopyranoside | Phenylpropanoids | C_26_H_34_O_11_*2-H | 1043.4130 | 1043.4100 | 2.9 | 359.1493 | Zuo et al., 2007 |

# REFERENCES

Chen, M., Gan, L., Lin, S., Wang, X., Li, L., Li, Y., et al. (2012). Alkaloids from the Root of *Isatis Indigotica*. *J. Nat. Prod.* 75, 1167–1176. doi:10.1021/ [np3002833](https://doi.org/10.1021/np3002833)

China Pharmaceutical University (2020), Traditional Chinese Medicine Systems Pharmacology Database and Analysis Platform. http://tcmspw.com/tcmsp.php.org/, 2020 [Accessed December 12, 2020]

Guo, Q., Sun, Y., Tang, Q., Zhang, H., and Cheng, Z. (2020). Isolation, Identiﬁcation, Biological Estimation, and Proﬁling of Glucosinolates in *Isatis Indigotica* Roots. *J. Liquid Chromatogr. Relat. Tech.* 43, 645–656. doi:10.1080/ 10826076.2020.1780605

He, L.-W., Li, X., Chen, J.-W., and Sun, D.-D. (2006a). Studies on Water-Soluble Chemical Constitutions in Radix Isatidis. *Chin. Pharm. J.* 17, 232–234. doi:10.3969/j.issn.1001-0408.2006.03.034

Kikuchi, M., and Kikuchi, M. (2005). Studies on the Constituents of Swertia Japonica MAKINO II. On the Structures of New Glycosides. *Chem. Pharm. Bull.* 53, 48–51. doi:10.1248/cpb.53.48

Kizil, S., Turk, M., Çakmak, Ö., Özgüven, M., and Khawar, K. M. (2009). Microelement Contents and Fatty Acid Compositions of Some Isatis Species Seeds. *Not. Bot. Horti. Agrobot. Cluj. Napoca.* 37, 175–178. doi:10.15835/ nbha3713115

Li, X. (2010). *Chemical Constituents and Quality Control of Radix Isatidis. [master*’*s Thesis]*. [Changzhi (Shanxi)]: Shanxi Medicinal University

Li, X., Chen, A.-J., and Li, C. (2010). Studies on Water-Soluble Chemical Constituent in Radix Isatidis. *Chin. J. Exp. Tradit. Med. Form.* 16, 64–67. doi:10.13422/j.cnki.syfjx.2010.05.037

Liu, S., Yan, J., Li, H.-L., Song, F.-R., Liu, Z.-Y., Liu, Z.-Q., et al. (2010). Studies on Chemical Constituents of Compound Indigowoad Root Granule by Mass Spectrometry. *Chem. J. Chin. Univ.* 31, 1137–1142. doi:10.1016/S1872- 2040(09)60019-0

Liu, S.-F., Zhang, Y.-Y., Zhou, L., Lin, B., Huang, X.-X., Wang, X.-B., et al. (2018). Alkaloids with Neuroprotective Effects from the Leaves of *Isatis Indigotica* Collected in the Anhui Province, China. *Phytochemistry* 149, 132–139. doi:10.1016/j.phytochem.2018.02.016

Liu, Y.-F., Chen, M.-H., Guo, Q.-L., Lin, S., Cheng., B.-X., Jiang, Y.-P., et al. (2015a). Antiviral Glycosidic Bisindole Alkaloids from the Roots of *Isatis Indigotica*. *J. Asian Nat. Prod. Res.* 17, 689–704. doi:10.1080/ 10286020.2015.1055729

Liu, Y.-F., Chen, M.-H., Lin, S., Li, Y.-H., Zhang, D., Jiang, J.-D., et al. (2015b). Indole Alkaloid Glucosides from the Roots of *Isatis Indigotica*. *J. Asian Nat. Prod. Res.* 18, 1–12. doi:10.1080/10286020.2015.1117452

Liu, Y.-F., Wang, X.-L., Chen, M.-H., Lin, S., Li, L., and Shi, J.-G. (2016). Three Pairs of Alkaloid Enantiomers from the Root of *Isatis indigotica*. *J*. *Acta Pharm Sin B.* 6, 141-147. doi: 10.1016/j.apsb.2016.01.003

Maugard, T., Enaud, E., Choisy, P., and Legoy, M. D. (2001). Identification of an Indigo Precursor from Leaves of *Isatis tinctoria* (Woad). *J*. *Phytochemistry.* 58, 897-904. doi: 10.1016/s0031-942200335-1

Meng, L., Guo, Q., Liu, Y., Chen, M., Li, Y., Jiang, J., et al. (2017a). Indole Alkaloid Sulfonic Acids from an Aqueous Extract of *Isatis Indigotica* Roots and Their Antiviral Activity. *Acta Pharmaceutica Sinica B* 7, 334–341. doi:10.1016/ j.apsb.2017.04.003

Meng, L.-J., Guo, Q.-L., Xu, C.-B., Zhu, C.-G., Liu, Y.-F., Chen, M.-H., et al. (2017b). Diglycosidic Indole Alkaloid Derivatives from an Aqueous Extract of *Isatis Indigotica* Roots. *J. Asian Nat. Prod. Res.* 19, 529–540. doi:10.1080/ 10286020.2017.1320547

Mohn, T., Cutting, B., Ernst, B., and Hamburger, M. (2007). Extraction and Analysis of Intact Glucosinolates-A Validated Pressurized Liquid Extraction/ liquid Chromatography-Mass Spectrometry Protocol for *Isatis Tinctoria*, and Qualitative Analysis of Other Cruciferous Plants. *J. Chromatogr. A* 1166, 142–151. doi:10.1016/j.chroma.2007.08.028

Pan, Y.-L. (2014). *Study the Chemical Composition of Effective Extraction of Isatidis Radix and its Composition Analysis of Different Regions. [master*’*s Thesis]*. [Nanjing (Jiangsu)]: Nanjing University of Traditional Chinese Medicine

Pan, Y.-L., Xue, P., Li, X., Chen, J. W., and Li, J. (2013). Determination of Nucleosides and Nucleobases in Isatidis Radix by HILIC-UPLC-MS/MS. *Anal. Methods*. 5, 6395–6400. doi:10.1039/C3AY40841H

Peng, S.-P., and Gu, Z.-L. (2005a). Recent Progress in the Studies of Chemical Constituents and Pharmacological Effects on Roots of *Isatis Indigotica*, *Chin*. *Wild Plant Res.* 24, 4–7. doi:10.3969/j.issn.1006-9690.2005.05.002

Peng, Y., Zhang, L.-P., Song, H., Pan, W.-S., and Sun, Y.-Q. (2005b). The Chemical Constituents from *Isatis Indigotica* Fort, I. *Chin. J. Med. Chem.* 15, 371–372. doi:10.14142/j.cnki.cn21-1313/r.2005.06.017

Sun, D.-D., He, L.-W., Li, X., Chen, J.-W., and Ding, L. (2007). Study on Chemical Constituents of Radix Isatidis. *Chin. Pharm. J.* 18, 172–173.

Sun, D., Dong, W., Li, X., and Zhang, H. (2009). Isolation, Structural Determination and Cytotoxic Activity of Two New Ceramides from the Root of *Isatis Indigotica*. *Sci. China Ser. B-chem.* 52, 621–625. doi:10.1007/ s11426-008-0146-9

The Metabolomics Innovation Centre (2020). *The Human Metabolome Database*. https://hmdb.ca/ [Accessed December 12, 2020]

Wang, R., Runco, J., Yang, L., Yu, K., Li, Y., Chen, R., et al. (2014). Qualitative and Quantitative Analyses of Goitrin-Epigoitrin in *Isatis Indigotica* Using Supercritical Fluid Chromatography-Photodiode Array Detector-Mass Spectrometry. *RSC Adv.* 4, 49257–49263. doi:10.1039/c4ra02705a

Wang, X.-L., Chen, M.-H., Wang, F., Pu, P.-B., Lin, S., Zhu, C.-G., et al. (2013). Chemical Constituents from Root of *Isatis Indigotica*. *China J. Chin. Mate. Med.* 38, 1172–1182. doi:10.4268/cjcmm2013081210.1080/03632415.2013.848346

Waters (2020), Waters UNIFI Scientific Information System. https://www.waters.com.org/, [Accessed December 12, 2020]

Wei, X.-Y., Leung, C.-Y., Wong, C. K. C., Shen, X.-L., Wong, R. N. S., Cai, Z.-W., et al. (2005). Bisindigotin, a TCDD Antagonist from the Chinese Medicinal Herb *Isatis Indigotica*. *J. Nat. Prod.* 68, 427–429. doi:10.1021/np049662i

Wu, X., Qin, G., Cheung, K.-K., and Cheng, K.-F. (1997). New Alkaloids from *Isatis Indigotica*. *Tetrahedron.* 53, 13323–13328. doi:10.1016/S0040-4020(97)00846-6

Xiao, S.-S., Bi, K.-S. and Sun, Y.-Q. (2007). Identiﬁcation of Chemical Constituents in the Root of *Isatis Indigotica* Fort. by LC/DAD/ESI/MS/MS. *J. Liquid Chromatogr. Relat. Tech.* 30, 73–85. doi:10.1080/10826070601034295

Yang, L., Jiang, H., Wang, G., Wang, M., Ding, L., Chen, L., et al. (2014a). Phenylpropanoids and Some Nitrogen-Containing Constituents from the Roots of *Isatis Indigotica* Fort. (Cruciferae). *Biochem. Syst. Ecol.* 54, 313–315. doi:10.1016/j.bse.2014.03.004

Yang, L., Wang, G., Wang, M., Jiang, H., Chen, L., Zhao, F., et al. (2014b). Indole Alkaloids from the Roots of *Isatis Indigotica* and Their Inhibitory Effects on Nitric Oxide Production. *Fitoterapia*. 95, 175–181. doi:10.1016/j.ﬁtote.2014.03.019

Zhang, D.-D., Li, Q.-Y., Shi, H.-Y., Chen, K.-X., Li, Y.-M., and Wang, R. (2019a).Glycosides from Roots of *Isatis Indigotica*. *Chin. Tradit. Herb. Drugs*. 50, 3575–3588. doi:10.7501/j.issn.0253-2670.2019.15.009

Zhang, D., Ruan, D., Li, J., Chen, Z., Zhu, W., Guo, F., et al. (2020a). Four Undescribed Sulfur-Containing Indole Alkaloids with Nitric Oxide Inhibitory Activities from *Isatis Tinctoria* L. Roots. *Phytochemistry.* [doi:10.1016/j.phytochem.2020.112337](https://doi.org/10.1016/j.phytochem.2020.112337)

Zhang, D.-D., Ruan, D.-Q., Li, Q,-Y., Chen, K.-X., Li, Y.-M., and Wang, R. (2020b). Study on Alkaloids from Alcohol Extract of Radix Isatidis. *CJTCMP*. 35, 2287–2291. CNKI:SUN:BXYY.0.2020-05-018

Zhang, D.-D, Sun, Y., Chen, Z.-Q., Jia, Q., Zhu, W. L., Chen, K.-X., et al. (2020c). Bisindole Alkaloids with Nitric Oxide Inhibitory Activities from an Alcohol Extract of the *Isatis Indigotica* Roots. *Fitoterapia*. d[oi:10.1016/](https://doi.org/10.1016/j.fitote.2020.104654) [j.ﬁtote.2020.104654](https://doi.org/10.1016/j.fitote.2020.104654)

Zhao, N. (2015). *Study on the Serum Pharmacochemistry of Banlangen Based on the LC-MS. [master*’*s Thesis]*. [Haerbin (Heilongjiang)]: Heilongjiang University of Traditional Chinese Medicine

Zeng, J., Guo, Z., Xiao, Y., Wang, C., Zhang, X., and Liang, X. (2010). Puriﬁcation of Polar Compounds from Radix Isatidis Using Conventional C_18_ Column Coupled with Polarcopolymerized C_18_ Column. *J. Sep. Sci.* 33, 3341–3346. doi:10.1002/jssc.201000417

Zou, P., and Koh, H. L. (2007). Determination of Indican, Isatin, Indirubin and Indigotin in *Isatis Indigotica* by Liquid Chromatography/electrospray Ionization Tandem Mass Spectrometry. *Rapid Commun. Mass. Spectrom.* 21, 1239–1246. [doi:10.1002/rcm.2954](https://doi.org/10.1002/rcm.2954)

Zuo, L., Li, J.-B., Xu, J., et al. (2007). Studies on Chemical Constituents in Root of *Isatis indigotica*. *J. Chin. Mater. Med.* 32, 688–691
